# Shifting ranges and conservation challenges for lemurs in the face of climate change

**DOI:** 10.1002/ece3.1418

**Published:** 2015-02-17

**Authors:** Jason L Brown, Anne D Yoder

**Affiliations:** Biology Department, Duke UniversityDurham, North Carolina, 27705

**Keywords:** ANUSPLIN, BIOMOD, ecological niche modeling, ensemble, least-cost corridors, Madagascar, micro-endemism, pseudo-absence selection, species distribution modeling, Strepsirrhini

## Abstract

Geospatial modeling is one of the most powerful tools available to conservation biologists for estimating current species ranges of Earth's biodiversity. Now, with the advantage of predictive climate models, these methods can be deployed for understanding future impacts on threatened biota. Here, we employ predictive modeling under a conservative estimate of future climate change to examine impacts on the future abundance and geographic distributions of Malagasy lemurs. Using distribution data from the primary literature, we employed ensemble species distribution models and geospatial analyses to predict future changes in species distributions. Current species distribution models (SDMs) were created within the BIOMOD2 framework that capitalizes on ten widely used modeling techniques. Future and current SDMs were then subtracted from each other, and areas of contraction, expansion, and stability were calculated. Model overprediction is a common issue associated Malagasy taxa. Accordingly, we introduce novel methods for incorporating biological data on dispersal potential to better inform the selection of pseudo-absence points. This study predicts that 60% of the 57 species examined will experience a considerable range of reductions in the next seventy years *entirely* due to future climate change. Of these species, range sizes are predicted to decrease by an average of 59.6%. Nine lemur species (16%) are predicted to expand their ranges, and 13 species (22.8%) distribution sizes were predicted to be stable through time. Species ranges will experience severe shifts, typically contractions, and for the majority of lemur species, geographic distributions will be considerably altered. We identify three areas in dire need of protection, concluding that strategically managed forest corridors must be a key component of lemur and other biodiversity conservation strategies. This recommendation is all the more urgent given that the results presented here do not take into account patterns of ongoing habitat destruction relating to human activities.

## Introduction

Climate change is a looming threat to Earth's biodiversity, with island ecosystems being among the most gravely threatened (Wetzel et al. 2012, 2013; Gibson et al. 2013). Entire faunal assemblages may perish as a consequence (Ricketts et al. 2005; Pounds et al. 2006; Moritz and Agudo 2013). Geospatial analyses can be utilized to forecast the directionality and magnitude of shifting habitats and thus be deployed for predicting the future distribution of biodiversity. Conservation biologists and affected governing authorities can then reference these predictions to implement proactive strategies (Heller and Zavaleta 2009; Rakotomanana et al. 2013). Although the lemurs of Madagascar have been identified as the world's most endangered vertebrates (Schwitzer et al. 2013), it is unknown how climate change will impact their future distributions and already high-risk status. For example, lemur reproduction has been linked with climatic variability (Dunham et al. 2011), and climatic changes may be an additional threat. To look at the potential distribution changes resulting from future climate change, we employ ensemble species distribution models (Araujo and New 2007) under a conservative model (IPCC 2007) to assess the impact of climate change on future abundance and distributions of 57 lemur species. The predictive power of these analyses is enhanced by novel methods derived here for the incorporation of biological data to better inform the selection of pseudo-absence points. The results reveal a complex mixture of future habitat stability and contraction. We identify three areas in dire need of protection; each with different levels of current forestation and governmental protection, and each with differing climatic and ecological profiles. Most notably, the results of this study indicate that ranges will shift for the majority of lemur species, in certain cases, by hundreds of kilometers. It is therefore imperative for the long-term survival of lemurs that conservation strategies be implemented for the establishment and protection of forest corridors. These corridors will provide the means for lemur (and other) species to follow suitable habitats as they shift in response to a changing global climate (Moilanen et al. 2009).

In July 2012, the International Union for Conservation of Nature (IUCN) species survival group concluded that lemurs are the most endangered group of vertebrates on Earth, with threat levels exceeding those of all other mammals, amphibians, birds (including reptiles), and boney fishes (Schwitzer et al. 2013). Of the 103 species recognized by the IUCN's Red List of Threatened Species, 94% are threatened (24 as critically endangered, 49 as endangered, and 20 vulnerable)(Schwitzer et al. 2013). The ever-worsening status is associated with numerous human pressures including population growth and extreme poverty, introduced invasive species, illegal logging, mining, subsistence farming, and increased pressures from poaching and the bushmeat trade (Barrett and Ratsimbazafy 2009; Barrett et al. 2010; Rakotomanana et al. 2013). Approximately 80% of Madagascar's population lives in rural areas and depends on subsistence agriculture. In the last two centuries, deforestation has claimed more than 90% of the island's natural habitats (Ingram and Dawson 2005; Harper et al. 2007). The remaining unprotected forests are under unrelenting pressure from the slash-and-burn cultivation of rice (Erdmann 2003) as well as from the collection of lumber for cooking and construction.

Annually, the Malagasy consume 22,000,000 m^3^ of wood (Rabenandrasana 2007). Given that the current population of 21.9 million human inhabitants is projected to grow to 53.6 million by the year 2050 (Rakotomanana et al. 2013), which will result in increased pressure on Madagascar's natural habitats, the need for targeted conservation action is urgent requiring both short- and long-term planning. These pressures place the survival of lemurs and other members of Madagascar's highly endemic biota under grave threat. Climate change has been recognized as one of the most important determinants for changing species distributions (Araujo and Rahbek 2006; Parmesan 2006; Beaumont et al. 2007), with regional changes in precipitation and temperature being especially potent forces (Peters and Darling 1985; Parmesan 1996; Hannah et al. 2008; Busch et al. 2012). Several studies have demonstrated that species are already experiencing distribution shifts toward the poles or to higher elevations, with some species driven to extinction by rapid climate change (Parmesan and Yohe 2003; Root et al. 2003). Such global-scale studies have been expanding rapidly over the past several decades. More recently, there has been a call for a focus on smaller and more rapid assessments of “pressing questions that have a particular political interest and for which science is evolving quickly” (Editoral 2013). This call is especially relevant to the conservation crisis currently facing Madagascar's highly threatened endemic biota. Conservation planning in Madagascar has prioritized areas that include a network of habitats containing as many endemic species as possible (Kremen et al. 2008). In August 2013, the IUCN published a comprehensive lemur conservation action plan that aimed to maximize the immediate survival of lemurs by outlining management goals for the next 3 years (Schwitzer et al. 2013). These measures, however, do not take into account the directionality, magnitude or probability of species’ future range shifts in response to a changing climate. Many populations can respond by shifting from altered and unsuitable habitats to more favorable adjacent habitats, although such range shifts are only feasible if suitable intervening habitats persist and are accessible.

Here, we aim to estimate the distributional changes of lemurs in response to climate change. We identify and characterize key areas of species richness and endemism in present-day Madagascar, comparing present estimates to those projected to occur 70 years from now (ca. year 2080). On the basis of these comparisons, we identify areas of key conservation priority required to maximize the persistence of lemur species, and by extension, the myriad endemic biodiversity with which they share their habitat.

## Methods

### Species occurrence data

We compiled 4600 occurrence localities on all species of lemurs from the primary literature (see Table S2 for details) and from the national database, REBIOMA, a database with expertly vetted occurrence data (http://data.rebioma.net). Only species with ≥6 unique occurrence points were modeled (Pearson et al. 2007; Kramer-Schadt et al. 2013); the final data set represented 57 lemur species sampled throughout Madagascar with 6–133 unique localities per species. These occurrence data contained at least one species from every genus of extant lemur (see Table S2) and were vetted by experts for accuracy. Spatial sampling biases were corrected by rarefying the data, randomly selecting a single occurrence point when many are present within a shared area, at a 5 km^2^ spatial resolution for non-micro-endemic species (with minimum convex polygons ≥2500 km^2^)( Peterson et al. 2011; Kramer-Schadt et al. 2013).

### Climate data

The current and future climate data were comprised of the original 19 standard Bioclim variables [30 arc-sec resolution, from worldclim.org (Hijmans et al. 2005) and ICP4.org (IPCC 2007)] and 16 additional Bioclim variables (variables 20–36, 2.5 arc-min resolution) pertaining to soil moisture and solar radiation [from the Climond.org data set (Kriticos et al. 2012)]. The 16 variables from Climond.org were only available at a coarser resolution (2.5 arc-min vs. 30 arc-sec) and were subsequently downscaled to 30 arc-sec using the ANUSPLIN method, a thin-plate smoothing method for noisy data (as follow by Hijmans et al. 2005). In brief, a high-resolution digital elevation model, latitude, and longitude were used as independent variables to predict local spatial relationships of the coarser climate data and used interpolate the climate data into higher spatial resolution (Hijmans et al. 2005). An additional independent variable, annual precipitation, was used for the downscaling of variables pertaining to solar radiation (Bioclim 20-27). The inclusion of annual precipitation allowed for dependences of solar radiation on cloud cover associated with rainfall (Hutchinson et al. 1984). Two additional independent variables, slope and aspect (in additional to elevation), were used to downscale the climate variables pertaining to soil moisture. These were included because both affect the amount of solar radiation that habitats receive and thus influence the soil moisture and water retention (Geroy et al. 2011). These methods were repeated for the Bioclim variables 20–36 for the current climate data and two 2080 global circulation models (all initially from Climong.org). The final downscaled variables are available for download at www.SDMtoolbox.org. All variables were assessed for co-correlation using a Pearson r correlation. Of the 36 Bioclim variables, 16 variables were co-correlated below 0.75 *r*^2^ and were subsequently used for species distribution modeling [Bio1–4, 6, 12–14, 18, 20, 22–26, 31; performed in SDMtoolbox v1.0 (Brown 2014)].

Climate change projections were obtained from the Intergovernmental Panel on Climate Change (IPCC 2007) and Climond.org (Kriticos et al. 2012). The two global circulation models used were CSIRO Mark 3.0 (CSIRO, Australia) and MIROC-H (Center for Climate Research, Japan). The two models covered the high and low of climate sensitivity at each emission scenario, reflecting the amount of global warming for a doubling of the atmospheric CO_2_ concentration compared with 1990 levels (CSIRO Mark 3.0: 2.11°C and MIROC-H: 4.13°C). We used the A1B emission scenario as it represents a moderate temperature change scenario. In brief, this model projects a future world of rapid economic growth, new and more efficient energy technologies, and convergence between regions (IPCC 2007). The A1B scenario adopts a balance across all energy sources (fossil and renewable) for the technological change in the energy system (IPCC 2007). This scenario has been extensively used (van der Linden and Mitchell 2009) and represents a medium emission trajectory and results in midrange estimates of average global changes (IPCC 2007). Lastly, the Bioclim variables 20–36 were available for both current and future scenarios (Kriticos et al. 2012), thus making them directly comparable.

### Species distribution models

**Figure 1:**
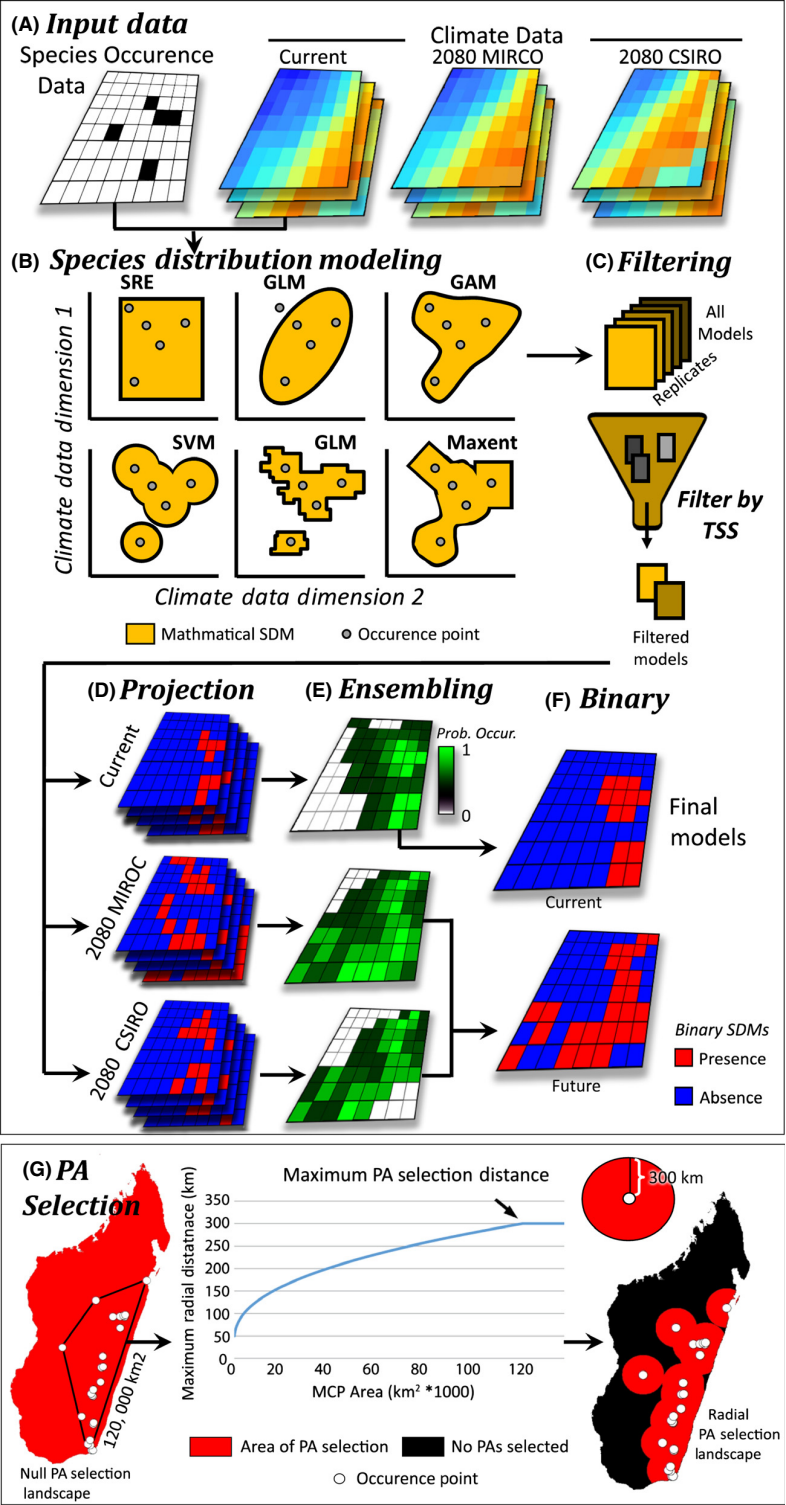
Overview of species distribution modeling here employed. (A) Species occurrence data and climate data were prepared for Madagascar, (B) SDMs were built based on ten widely used modeling techniques (only six are pictured). (C) The resulting models, including replicates of each method, are filtered based on their abilities to predict known occurrences and pseudo-absences using a true skill statistic (TSS). (D) The resulting models with TTS values ≥ 0.85 were projected throughout the climate of the current landscape and two future climate models. (E) Models for each scenario are compiled and compared, creating an ensemble probabilistic density landscape depicting the probability of a species occurrence throughout the landscape. (F) The ensemble models were then converted to a binary “presence/absence” landscape from which all geospatial analyses were performed on. (G) To create high-quality species distribution models, we carefully selected pseudo-absences (PAs). Here, we select PAs within a variable distance from each known occurrence point determined by calculating the area of a minimum convex polygon from occurrence points and transforming that to reflect a logistic curve with values from 50 to 300 km. This method is sensitive to changes in smaller ranges, making it suitable for use on micro-endemic to broadly distributed species.

Current species distribution models (SDMs) were created within the BIOMOD2 framework (Thuiller et al. 2009) (R package Biomod2). BIOMOD2 is a SDM method that capitalizes on ten widely used modeling techniques: artificial neural networks (ANN)(Ripley 1996), classification tree analysis (CTA)(Breiman 1984), generalized additive models (GAM)(Hastie et al. 1994), generalized linear models (GLM)(McCullagh and Nelder 1989), generalized boosted models (GBM, also known as boosted regression trees)(Ridgeway 1999), flexible discriminant analysis (FDA)(Hastie et al. 1994), multivariate adaptive regression splines (MARS)(Friedman 1991), Breiman and Cutler's random forest for classification and regression (RF)(Breiman 2001), surface range envelope (SRE, a.k.a. BIOCLIM)(Busby 1991), and maximum entropy (MaxEnt)(Phillips et al. 2006). BIOMOD2 accounts for intermodel variability by fitting ensembles of forecasts by simulating across more than one set of initial conditions, model classes, model parameters, and boundary conditions (see Araujo and New 2007 for a review). BIOMOD2 analyzes the resulting range of uncertainties with regard to area bounded by models, predictive consensus, and probabilistic density functions summarizing the likelihood of a species presence estimated from a large ensemble of SDMs (Araujo and New 2007; Thuiller 2007). In lemur species with a number of unique occurrences points >10, model calibration was performed on a random sample of the data (75%), and model evaluation was carried out on the remaining 25% with the true skill statistic (TSS) (10 replicates of each species). For species with 6–9 occurrences (*n* = 9), localities were jackknifed to evaluate and build the model. After the individual models were built, they were ensembled and converted to binary models based on a TSS cutoff of 0.85. Each ensemble model was proofed by a taxonomic expert. Based on the optimal of number pseudo-absences (PAs) required, the 10 modeling techniques were placed in three classes (high PAs, mid PAs, low PAs) and ensemble modeling was performed for each class (see below for information on the number PAs for each class)(Barbet-Massin et al. 2012). Each PA class produced a single, continuous probabilistic density ensemble model that was then weighted by the number of models included. The three weighted layers were summed to produce the final continuous ensemble model for each climate scenario. For each PA class final, binary ensemble models were summed and the product was divided by the number of classes contributing to each model to produce the final model. For the two SDMs based on the future global circulation models, ensemble models were averaged prior to conversion to a binary model. The final ensemble SDM was converted to a binary model by classifying values greater than (or equal to) 0.5 to a value of 1 and those lower to a value of 0. All final binary models (both current and future) were then clipped by areas of natural vegetation in 2005 (ONE 2009). This model represented the predicted binary distribution of each species (see Fig.1 for an overview of methods).

To minimize future predictions in habitats that could not be feasibly dispersed into by the year 2080, a maximum dispersal limit of 100 km (ca. 1.4 km/year) was enforced from areas of “suitable habitat” in contemporary distributions (Anderson 2013; performed in SDMtoolbox v1.0, Brown 2014). Future nonanalogous climates (FNAC) were also identified, and inferences from models projected into these areas were interpreted with caution (see Fig. S3), particularly if ranges were predicted to expand to these environments (vs. predictions of stability or decline). Due to uncertainty of species’ responses under such scenarios, no conservation recommendations were based on species’ expansions or contractions into geographic areas with FNAC. However, predictions of stability in FNAC helped to determine the second priority area (habitats surrounding the Mangoky River). Not surprisingly (due to persisting suitability), the nonanalogous variables in this area (identified by running separate MESS plots in MaxEnt) were not central to predicting habitat suitability for these species.

### Pseudo-absence selection

Model overprediction is a common issue associated with models derived for Malagasy taxa. This is, in large part, due to the physiognomy of Madagascar that extends latitudinally (12–16°S) with three mountain massifs extending down the spine. The country is much narrower longitudinally (situated diagonally across 43–51°E) and a rain-shadow effect produces an arid western versant (Goodman and Benstead 2003). These factors create steep environmental gradients longitudinally, and many species’ distributions extend latitudinally across suitable ecotones. This type of physiognomy, coupled with areas of considerable topographic heterogeneity, is especially problematic for model evaluation and for selection of appropriate pseudo-absences.

Pseudo-absences (PAs) are meant to be compared with the presence data and help differentiate the environmental conditions under which a species can potentially occur. Typically, PAs are selected within a large rectilinear area, within this area there often exists habitat that is environmentally suitable, but was never colonized. When background points are selected within these habitats, this increases commission errors (false positives). As a result, the “best” performing model tends to be overfit because selection criterion favor a model that fails to predict the species in the climatically suitable, uncolonized habitats (Anderson & Raza, 2010; Barbet-Massin et al. 2012). The likelihood that suitable unoccupied habitats are included in background sampling increases with Euclidian distance from the species’ realized range. Thus, a larger study spatial extent can lead to the selection of a higher proportion of less informative background points (Barbet-Massin et al. 2012).

To circumvent this problem, many researchers have begun using PA selection methods that are more regional (VanDerWal et al. 2009). One common method constitutes sampling PAs within a maximum radial distance of known occurrences (Thuiller et al. 2009). However, selection of the maximum distance parameter can be difficult to choose and should reflect a realistic dispersal distance (e.g., habitat that is potential reachable through evolutionary time). Here, we selected the maximum radial search distance (MRSD) by calculating the area of a minimum convex polygon (or minimum convex polygon area, MCPA) from occurrence points and transforming them to reflect a logistic curve with MRSD values from 50 to 300 km. This method is more sensitive to changes in smaller range sizes. For example, it will apply a MRSD of 50, 100, and 200 km to species with MCPA of 100, 4000, and 40,000 km^2^, respectively (see equation in Box 1). Based on the optimal number of pseudo-absences (PAs) required, the 10 modeling techniques were placed in three classes: high PAs (GAM, GLM, SRE, MaxEnt, and ANN), mid PAs (MARs and FDA), and low PAs (CTA, GBM, RF). For the high PAs class, 10,000 PAs were used for all models (Barbet-Massin et al. 2012). In the mid and low PAs classes, the number of PAs equaled the number of unique occurrence points multiplied by 10 and 4, respectively (average, min–max: 324, 60–1330, and 130, 24–532) (Barbet-Massin et al. 2012).

**Box 1 Pseudo-absence maximum radial search distance equation**

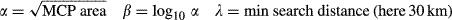


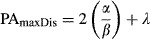


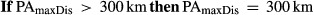

### Geospatial analyses

To measure the predicted distribution changes for each species, the binary SDMs were projected to Africa Albers Equal-Area Cylindrical projection in ArcMap 10.1 (ESRI 2012) at a spatial resolution of 0.9109 km^2^. The final binary models were then clipped by areas of natural vegetation in 2005 (ONE 2009) that limited species’ current distributions to areas of natural vegetation.

The future and current SDMs were then subtracted from each other, and areas of contraction, expansion, and stability were calculated. Each of these individual classes was summed separately for all species, generating a map displaying the intensity of contraction, expansion, and stability throughout Madagascar. Expansion was classified as a species that expanded its future range to more than 125% of their current predicted range, whereas contraction was classified as a species with a future range of 75% or less of their current predicted distributions. Stable species are those with future range areas between 75 and 125% of their current predicted distributions. To examine whether range shifts are predicted to occur, we calculated and connected the geographic centroids of the current and future binary SDMs in ArcGIS 10.1 using SDMtoolbox v1.0 (Brown 2014).

At the species level, expansion, stability, and contraction were also classified as a change in future range size of: >125, 125–75, and 75–0%, respectively. Core range shifts were calculated by measuring and connecting the geographic centroids of the current and future binary SDMs. To characterize areas of conservation priority, we measured the following: (1) the density of overlapping core range shift vectors to identify areas that are key to future dispersal and (2) areas of high species richness and high levels of micro-endemism for current and future (2080) periods. To calculate species richness, we summed the binary SDMs. To calculate areas of high micro-endemism, we calculated the weighted endemism, which is the sum of the reciprocal of the total number of cells each species in a grid cell is found within (Crisp et al. 2001). A weighted endemism emphasizes areas that have a high proportion of species with restricted ranges. We summed the weighted endemism values in a 10 × 10 neighborhood (ca. 10 km2) across Madagascar to extend the comparisons of regions with high levels of micro-endemism. Each of these metrics was standardized from 0 to 1, with values of 1 representing the highest values of biodiversity metrics for both time periods (current and future). Layers were then summed, output GIS calculation depicts areas of high species richness and micro-endemism through time (referred to as the HSRME layer).

Lastly, because distributions were predicted to change for many lemur species, we estimated areas of high dispersal importance for the species included here. Using the vectors of centroid changes, we calculated the density of overlapping vectors. This was performed in ArcGIS 10.1 using the line density function with a search radius of 50 km (ESRI 2012). Resulting areas of high line densities reflected areas that are hypothesized to be essential for future dispersal into suitable habitats. On the basis of these areas, we calculated least-cost corridors (categories of paths that include paths with slightly less suitable habitats relative to the optimum path) between currently protected areas to identify additional areas in need of protection. As a friction layer (the layer used to depict the site-by-site connectivity cost in least-cost corridor analyses), we inverted the HSRME layer and standardized it from 0 to 1, placing a friction value of 10 on deforested areas. Least-cost corridors were estimated in ArcGIS 10.1 using SDMtoolbox v1.0 using the default settings (Brown 2014).

## Results

We created high-performing species distribution models for 57 species of lemurs, which were then used to generate ensemble models of each species. Model performance was based on true skill statistic (TSS) score of ≥0.85. True skill statistic scores range from −1 to 1, with 0 indicating predictive no ability and 1 depicting a perfect ability to distinguish actual suitable and unsuitable habitat. Our results reveal that the majority of species (35 spp., 59.6%) are predicted to experience range contractions in future climates. Of these species, range sizes are predicted to decrease by an average of 67.1%. Of the 57 species modeled, 27 species (47%) have future distributions <50% of their current sizes; 14 species (25%) have distributions <20% of their current size; and six species (11%) are predicted to have distributions <1% of their current sizes. This includes three species predicted to go extinct (*Lepilemur microdon, Lepilemur hubbardorum,* and *Microcebus danfossi*, Table S1). Nine lemur species (16%) are predicted to expand their ranges in the future (Table S1). Of those species, on average, their ranges are predicted to expand by 180.2%. Only 13 species’ (22.8%) distribution sizes were predicted to be stable through time with an average range area of 99.6% of current sizes (Table 1).

**Figure 2:**
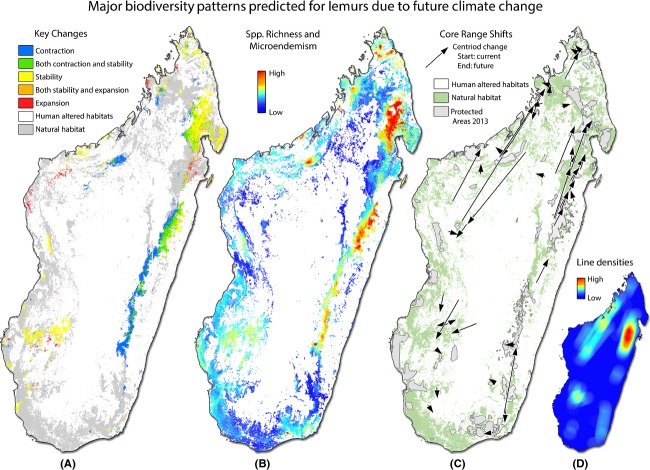
Major distribution patterns predicted for lemurs resulting from future climate change. (A) Depicts areas where three or more species will experience contraction, expansion, or stability. Cool colors correspond to areas of contraction and warmer colors depict areas of stability and expansion. (B) Depicts areas of high species richness and/or high levels of micro-endemism for both current and future (2080) scenarios. Warm colors depict areas with high biodiversity values at both time periods and should thus have the greatest conservation potential. (C) Map of core range shifts depicts the predicted distribution changes (based on the centers of their distributions) of each focal species. Each line depicts predicted distributional shifts of the species range centroid from current (start of arrow) to 2080 (end of arrow) scenarios. (D) Areas of highest conservation concern. The line densities depicted by warmer colors illustrate areas of high overlap in core range shifts through time. In these areas, it will be vital that protected areas are connected to facilitate dispersal into appropriate habitats.

**Figure 3:**
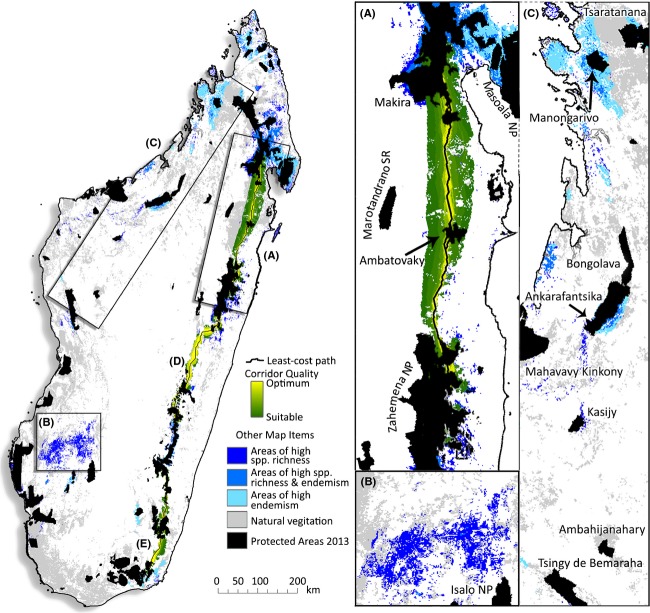
Conservation priority areas. Three regions of Madagascar with highest priority for protection and conservation of lemurs and their cohabitants. (A) Area 1 of highest conservation concern. Core range shift analyses suggest that many of the east-central populations may need to disperse northward via the forests between Zahamena NP, Ambatovaky, and Makira. Using least-cost corridors and estimates of high current and future species richness and micro-endemism, we identify a series of corridors that connect these regions – traversing the habitat of the highest suitability and ecological stability for the highest number of species. (B) Second priority area surrounding the Mangoky River. This area is predicted to be central in facilitating dispersal among southwestern ecotones, but also a region predicted to act as a sanctuary for many lemur species. (C) Third priority region identified is a wide range habitat in the NW. This region has experienced widespread deforestation and a stepping-stone reserve network combined with selective reforestation might be feasible. Two specific areas with high levels of micro-endemism in this region are the coastal forests near Ambaliha and Antsirabe (situated west of Manongarivo) and the coastal forests around the village of Mariarano (west of Bongolava). See Fig. S2 for information regarding D and E.

Our study identified three regions of Madagascar as the highest priorities for protection and conservation consequent to predictions of climate change. First, our analyses of core range shifts predict a large number of distributional shifts from east-central rainforests northward into the Masoloa peninsula (Fig.2C,D). These shifts coincide with a large number of lemur species predicted to experience range contractions in the east-central rainforests and the increased level of stability predicted in the Masoloa peninsula (Fig.2A). Using least-cost corridors and our estimates of high current and future species richness and micro-endemism, we identified a series of corridors that connect these regions, traversing the habitats of highest suitability and ecological stability for the largest number of species (Fig.3A).

The second priority region is the area surrounding the Mangoky River in the southwest Madagascar (Fig.3B). This area is not only predicted to be central in facilitating dispersal for many species among southwestern ecotones, but is also predicted to act as a sanctuary for many lemur species. This area is expected to experience nonanalogous climates by the year 2080, although even in light of this departure from observed climates in Madagascar, important environmental parameters necessary to sustain species are predicted to persist. The third priority region identified comprises a wide range of habitats in the northwest (Fig.3C), a region that has experienced widespread deforestation and habitat alteration. Consequently, a continuous NS corridor is not currently feasible, although a stepping-stone reserve network combined with selective reforestation might offer a solution. The forests immediately west of Manongarivo (Fig.3C) and the coastal forests around the village of Mariarano (west of Bongolava in Fig.3C) are predicted to possess high levels of micro-endemism and should be immediately assessed and considered for protection.

## Discussion

Ensemble species distribution models account for intermodel variability by fitting a group of species’ distribution models (SDMs) across more than one set of initial conditions: model classes, model parameters, and boundary conditions (see Araujo and New 2007 for a review). The use of a large ensemble from many SDMs, the uncertainties in the area bounded by the models, predictive consensus, and the probabilistic likelihood of a species occurrence are minimized (Araujo and New 2007; Thuiller 2007); and therefore, the final model is a conservative agreement of input parameter space and modeling algorithms. Here, in addition to the more commonly used temperature and precipitation climate data, we incorporated climate data on soil moisture and solar radiation data into our models. The resulting predictions therefore integrate the most detailed climatic, ecological, and biological data applied so far toward understanding the current status of lemur distributions and their probable fluctuations in response to climate change. These data are critical for making informed decisions pertaining to habitat protection with the goal of conserving lemurs and other members of Madagascar's irreplaceable biota.

The IUCN Red List shows that 94% of lemur species are currently threatened. Our study predicts that 60% of the 57 species modeled by in this analysis will experience considerable range reductions in the next 70 years *entirely* due to future climate change. Thus, the conservation impacts of these predictions should be considered highly conservative in that they ignore the progressive and ongoing effects of human-mediated habitat destruction. In context of spatial patterns, our estimates predict a large area of range contraction, >600 km in length, for up to 12 species of lemurs in the mid-elevation eastern rainforests. Other areas also are predicted to experience a large reduction in lemur species’ ranges, for example, around Ankarafantsika in the northwest (Fig.2A). Although our predictions are more extreme than estimates of range reductions of 11–27% that are based solely on models of vegetative change (Malcolm et al. 2006), they are nonetheless conservative in that they do not include many micro-endemic species due to limited locality data available for modeling. Given that species with small distributions typically possess narrower ecological tolerances, and even slight environmental changes can dramatically affect those species (Murray et al. 2011), future consideration of micro-endemics may reveal even greater threats.

Our predictions and recommendations are based on hypothesized trends of species’ range shifts with uncertainties minimized by the use of the ensemble modeling framework. Other factors impact species and their habitats (i.e., sociopolitical factors, biotic interactions among native species, or novel invasive species and diseases; e.g., see Chapman et al. 2014), but these are not explicitly included in our models. For example, climate change will also affect human populations in Madagascar – this includes changes in agricultural practices or tracking suitable agricultural sites. Even in light of these volatile extrinsic factors, many studies have demonstrated that climate-based model predictions can be highly accurate for anticipating range-shifting species (e.g., Morin and Thuiller 2009; Anderson 2013).

The most significant result to emerge from this study is the prediction that a majority of lemur species will experience range shifts – mostly contractions, in some cases by hundreds of kilometers. In many affected areas, suitable habitat does not exist between current and future distributions to allow for lemur species to traverse the landscape in pursuit of appropriate environments. To prepare for these contingencies, we urge that the conservation and local Malagasy governing communities place a priority on the establishment and maintenance of targeted forest corridors that will allow for these inevitable changes in lemur distributional ranges (Moilanen et al. 2009).

## Supporting Information

**Figure S1.** Spatial patterns of distributional changes. Warm colors depict higher values of each category; gray values depict human altered vegetation. A. Contraction, B. Stability, and C. Expansion of species distributions.

**Figure S2.** Conservation priorities (continued from Figure 2). E. A proposed corridor in southeastern Madagascar connecting Andohahala NP to Midongy Betotaka NP and then to Fandrina Vondrozo. D. A proposed corridor in east-central Madagascar connecting Ranomafana NP to Fadrina Vondrozo to Zahemena-Ankeniheny.

**Figure S3.** Shared areas of nonanalogous climate space in the two future climate scenarios. Here, more intense red indicates higher divergences from observed climate space and are climates that do not exist *anywhere* in contemporary Madagascar. The map, a MESS analysis, was generated in MaxEnt. The scatter plot depicts the first two PCs from single PCA analyses containing all three climate scenarios throughout Madagascar. The dotted areas are the major nonanalogue areas depicted in the map.

**Figure S4.** Lepilemur hubbardorum from Zombitse-Vohibasia National Park is one of three species predicted to be driven to extinction entirely due to future climate change, reports Brown & Yoder (2015). Photo: Jason L. Brown.

**Table S1.** Start region in core range shifts depicts area based on the subdivision of Madagascar into four units based on a grid as follows: NW=1, NE=2, SE=3, and SW=4. ICUN Red list status: CR = critically endangered; EN = endangered; VU = vulnerable; NT = near threatened; LC = least concern; and DD = data deficient.

**Table S2.** Table of occurrence localities used for this study and their sources.
