# Supplementary figures and images for: Shifting ranges and conservation challenges for lemurs in the face of climate change
Source: Ecol Evol. 2015 Feb 17;5(6):1131–42. doi: 10.1002/ece3.1418 (PMC4377258; doi:10.1002/ece3.1418)

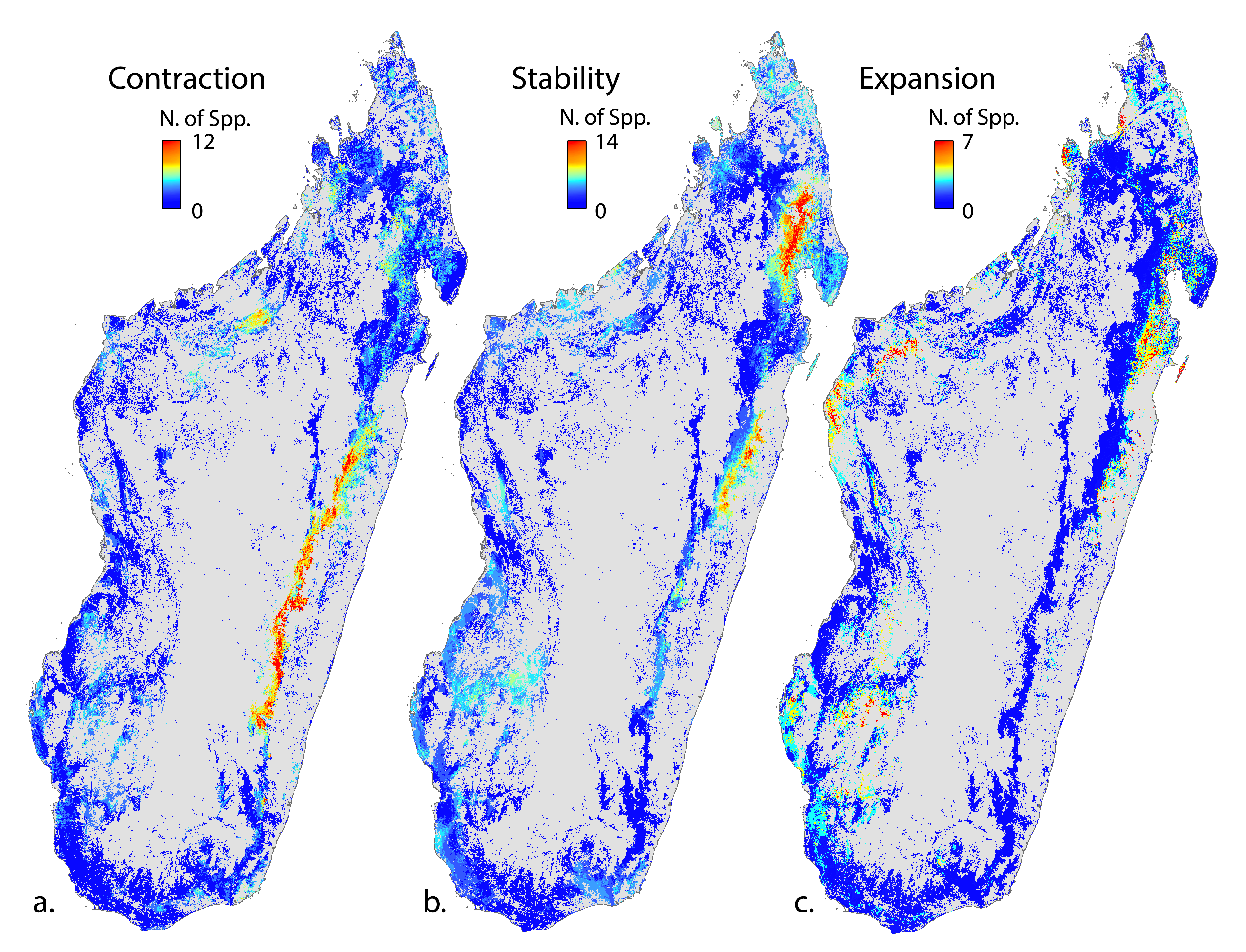

Supplement: Supplementary file 1 [file ece30005-1131-sd1.png]

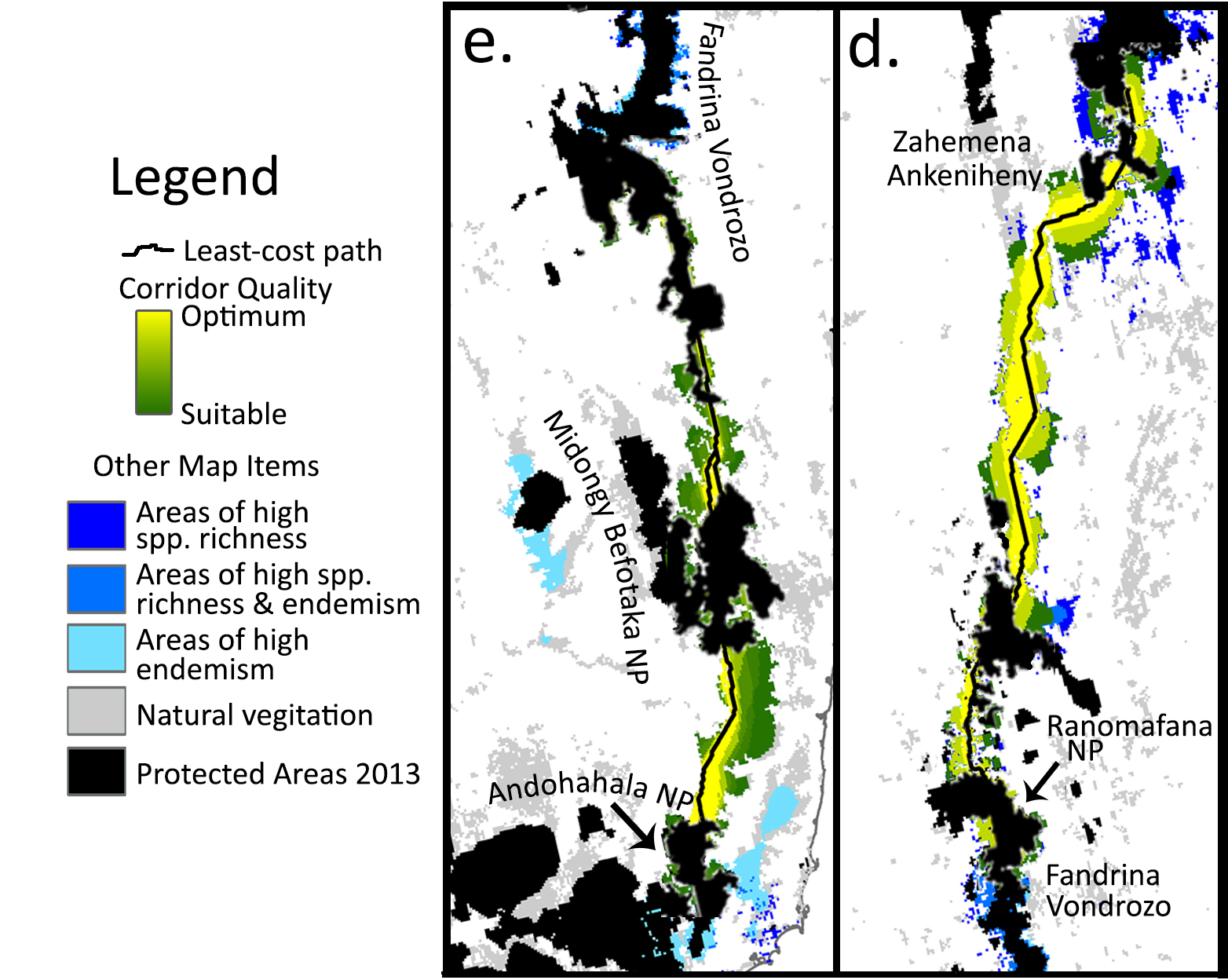

Supplement: Supplementary file 2 [file ece30005-1131-sd2.tif]

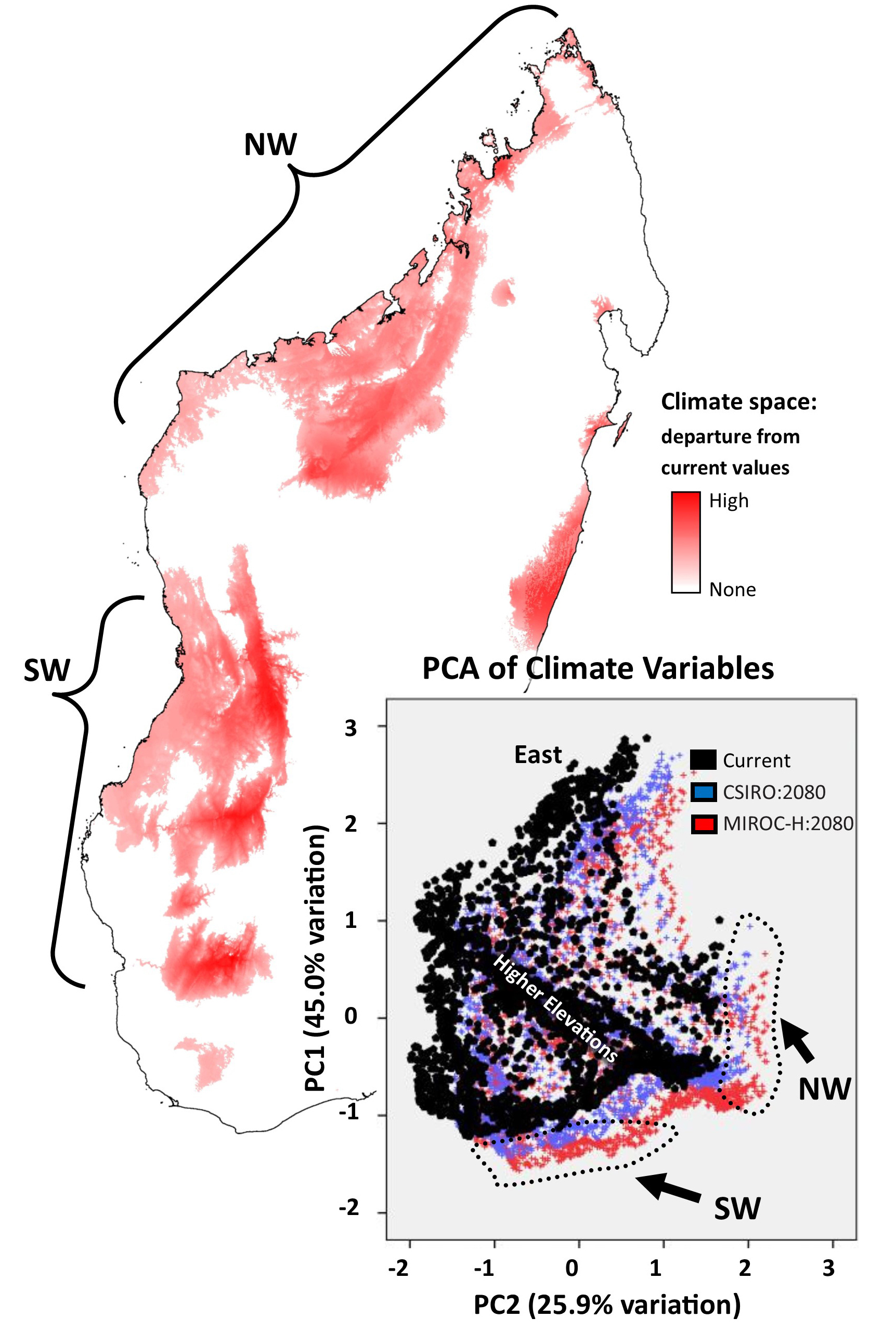

Supplement: Supplementary file 3 [file ece30005-1131-sd3.tif]

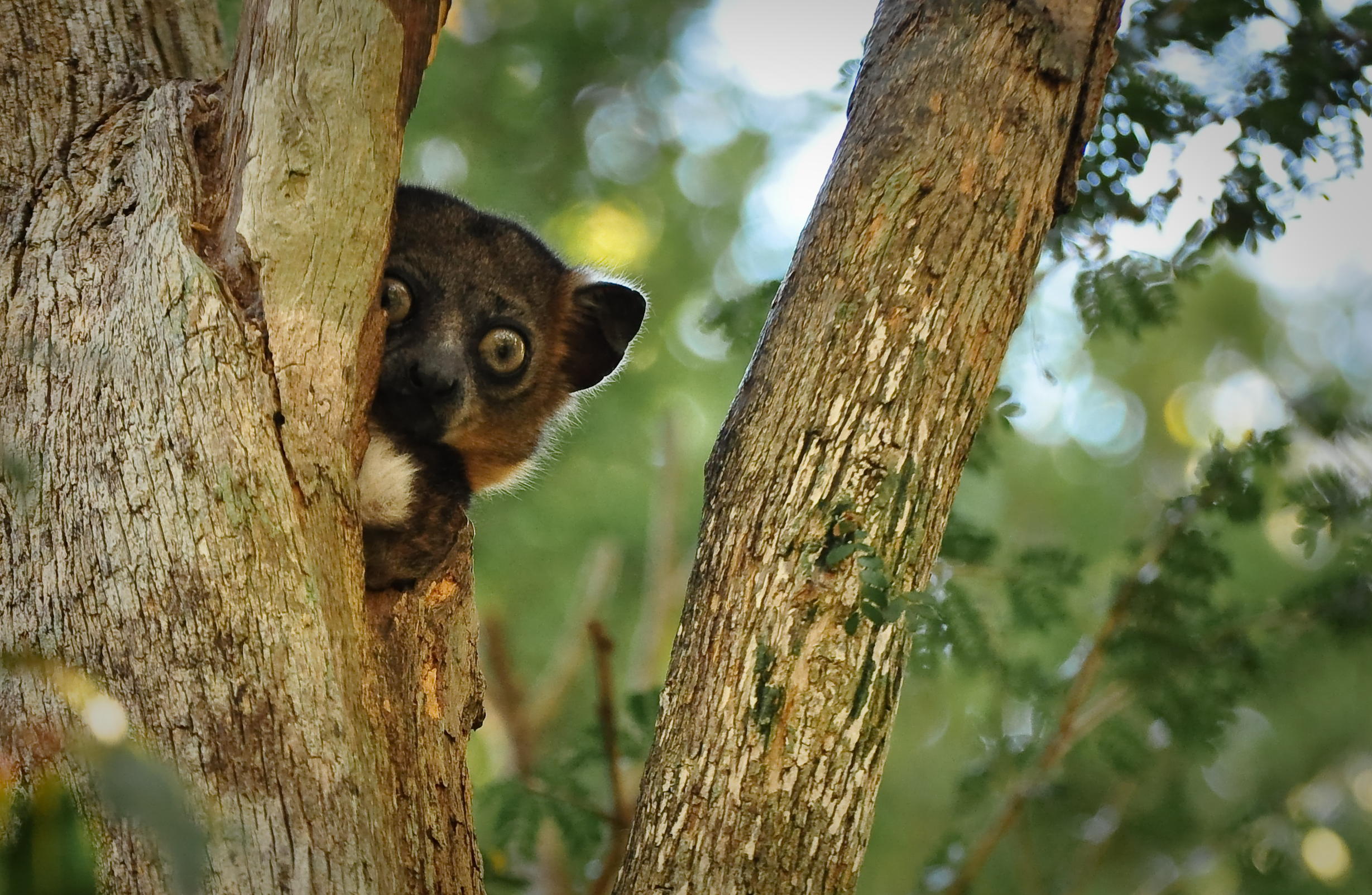

Supplement: Supplementary file 4 [file ece30005-1131-sd4.tif]
