# Supplementary material for: Shifting ranges and conservation challenges for lemurs in the face of climate change
Source: Ecol Evol. 2015 Feb 17;5(6):1131–42. doi: 10.1002/ece3.1418 (PMC4377258; doi:10.1002/ece3.1418)
Supplement: Supplementary file 5 [file ece30005-1131-sd5.docx]

**Supplemental Table 1.** Start region in core range shifts depicts area based on the subdivision of Madagascar into four units based on a grid as follows: NW=1, NE=2, SE=3 and SW=4. ICUN Red list status: CR= critically endangered; EN= endangered; VU= vulnerable; NT= near threatened; LC= least concern; and DD= data deficient.

|  | Total Distribution Area (km2) | | Distribution Changes Between 2000 and 2080 (km2) | | | Projected Area Change | | Core range shifts | IUCN Red List Status |
| --- | --- | --- | --- | --- | --- | --- | --- | --- | --- |
| Species | 2000 | 2080 | Contraction | Same | Expansion | km2 | % of Current | Km Direction/ Start Region | CR-EN-VU-NT-LC-DD |
| *Allocebus trichotis* | 13177.0 | 4697.6 | 8494.2 | 4682.7 | 14.9 | 8479.4 | 35.7% | 53.7 NE/2 | VU |
| *Avahi laniger* | 50584.0 | 363361.8 | 16445.6 | 34138.4 | 2223.4 | 18669.0 | 13.5% | 106.5 NE/2 | VU |
| *Avahi occidentalis* | 1126.8 | 247.4 | 879.5 | 247.4 | 0.0 | 879.5 | 22.0% | 21.8 NW/1 | EN |
| *Cheirogaleus major* | 25112.1 | 12235.5 | 15053.4 | 10058.7 | 2176.7 | 12876.6 | 48.7% | 144.0 NE/1 | DD |
| *Cheirogaleus medius* | 26725.4 | 14245.0 | 18696.1 | 8029.3 | 6215.8 | 12480.3 | 53.3% | 134.4 SW/2 | LC |
| *Daubentonia madagascariensis* | 31386.4 | 17672.4 | 14792.3 | 16594.1 | 1078.2 | 15870.5 | 7.3% | 60.2 NE/1 | EN |
| *Eulemur albifrons* | 16478.0 | 15170.0 | 1364.3 | 15113.7 | 56.3 | 1308.0 | 92.1% | 7.4 NE/1 | EN |
| *Eulemur cinereiceps* | 4244.2 | 14.1 | 4230.2 | 14.1 | 0.0 | 4230.2 | 0.3% | 140.8 NE/1 | CR |
| *Eulemur collaris* | 4195.4 | 1935.1 | 2262.8 | 1932.7 | 2.5 | 2260.3 | 46.1% | 55.0 SE/4 | EN |
| *Eulemur coronatus* | 2548.8 | 165.4 | 2393.4 | 155.439 | 10.0 | 2403.4 | 0.4% | 18.6 NW/1 | EN |
| *Eulemur flavifrons* | 760.3 | 88.5 | 675.1 | 85.2 | 3.3 | 671.8 | 11.6% | 18.3 SW/1 | CR |
| *Eulemur fulvus* | 19077.5 | 10158.8 | 9306.7 | 9770.8 | 388.0 | 8918.7 | 53.3% | 10.4 W/2 | NT |
| *Eulemur macaco* | 4115.2 | 2669.8 | 1682.0 | 2433.2 | 236.6 | 1445.4 | 64.9% | 14.9 W/2 | VU |
| *Eulemur rubriventer* | 21226.9 | 5067.4 | 16295.2 | 4931.7 | 135.7 | 16159.5 | 23.9% | 286.0 NE/2 | VU |
| *Eulemur rufifrons* | 21484.2 | 24921.8 | 3521.1 | 17963.1 | 6958.7 | -3437.6 | 116.0% | 67.5 W/3 | NT |
| *Eulemur rufus* | 16843.7 | 23952.2 | 4198.7 | 12645.0 | 11307.2 | -7108.5 | 142.2% | 251.5 SW/1 | VU |
| *Eulemur sanfordi* | 1401.5 | 651.9 | 757.0 | 644.5 | 7.4 | 749.6 | 46.5% | 6.4 NW/1 | EN |
| *Hapalemur aureus* | 2672.3 | 0.8 | 2671.5 | 0.8 | 0.0 | 2671.5 | 0.03% | 12.5 W/4 | CR |
| *Hapalemur griseus* | 23248.1 | 6646.0 | 17306.2 | 5941.9 | 704.1 | 16602.1 | 28.6% | 265.1 NE/2 | VU |
| *Hapalemur meridionalis* | 431.0 | 338.4 | 353.3 | 77.8 | 260.6 | 92.7 | 78.5% | 222.3 NE/4 | VU |
| *Hapalemur occidentalis* | 6901.6 | 104.2 | 6846.2 | 55.4 | 48.8 | 6797.4 | 1.5% | 588.9 NE/1 | VU |
| *Indri indri* | 15429.8 | 21528.1 | 1337.0 | 14092.8 | 7435.3 | -6098.3 | 139.5% | 30.5 NE/2 | CR |
| *Lemur catta* | 26325.0 | 16582.3 | 12031.1 | 14293.9 | 2288.4 | 9742.7 | 63.0% | 41.2 SE/3 | EN |
| *Lepilemur ankaranensis* | 834.0 | 781.8 | 59.6 | 774.4 | 7.4 | 52.1 | 93.8% | 1.3 NW/2 | EN |
| *Lepilemur dorsalis* | 2860.9 | 3395.4 | 183.7 | 2677.3 | 718.1 | -534.5 | 118.7% | 1.1 W/1 | VU |
| *Lepilemur edwardsi* | 5833.5 | 1859.8 | 3979.5 | 1854.1 | 5.8 | 3973.7 | 31.9% | 122.4 NE/2 | VU |
| *Lepilemur hubbardorum* | 437.7 | 0.0 | 437.7 | 0.0 | 0.0 | 437.7 | 0.0% | NA | EN |
| *Lepilemur leucopus* | 269.7 | 31.4 | 251.5 | 18.2 | 13.2 | 238.3 | 11.7% | 30.7 W/4 | EN |
| *Lepilemur microdon* | 984.5 | 0.0 | 984.5 | 0.0 | 0.0 | 984.5 | 0.0% | NA | EN |
| *Lepilemur mustelinus* | 27279.7 | 26438.3 | 8627.4 | 18652.3 | 7786.0 | 841.4 | 96.9% | 145.8 NE/3 | NT |
| *Lepilemur randrianasoloi* | 37741.4 | 4882.9 | 33044.6 | 4696.8 | 186.2 | 32858.4 | 12.9% | 228.9 SW/3 | EN |
| *Lepilemur seali* | 5349.5 | 4882.1 | 1283.2 | 4066.3 | 815.8 | 467.4 | 91.3% | 63.2 NE/2 | VU |
| *Lepilemur wrightae* | 489.0 | 128.2 | 360.7 | 128.2 | 0.0 | 360.7 | 26.2% | 7.2 W/4 | EN |
| *Microcebus arnholdi* | 746.3 | 1000.2 | 311.9 | 434.4 | 565.9 | -254.0 | 134.0% | 297.8 SW/1 | EN |
| *Microcebus danfossorum* | 766.9 | 0.0 | 766.9 | 0.0 | 0.0 | 766.9 | 0.0% | NA | DD |
| *Microcebus griseorufus* | 16411.8 | 26353.9 | 848.0 | 15563.8 | 10790.1 | -9942.1 | 160.6% | 36.9 SE/3 | LC |
| *Microcebus murinus* | 23717.2 | 14807.6 | 15438.1 | 8279.1 | 6528.5 | 8909.6 | 62.4% | 200.4 SW/3 | LC |
| *Microcebus myoxinus* | 4172.2 | 6229.8 | 0.0 | 4172.2 | 2057.6 | -2057.6 | 149.3% | 28.8 W/1 | VU |
| *Microcebus ravelobensis* | 1654.7 | 306.9 | 1347.7 | 306.9 | 0.0 | 1347.7 | 18.6% | 139.8 NE/1 | EN |
| *Microcebus rufus* | 21957.5 | 9190.9 | 14340.2 | 7617.3 | 1573.6 | 12766.6 | 41.9% | 91.3 NE/2 | VU |
| *Microcebus sambiranensis* | 1079.7 | 377.3 | 970.5 | 109.2 | 268.1 | 702.4 | 34.9% | 29.6 N/1 | EN |
| *Microcebus tavaratra* | 774.4 | 521.2 | 253.2 | 521.2 | 0.0 | 253.2 | 67.3% | 3.2 NW/1 | VU |
| *Mirza coquereli* | 6657.6 | 8551.3 | 983.7 | 5673.9 | 2877.5 | -1893.8 | 128.4% | 112.4 S/3 | EN |
| *Mirza zaza* | 4699.3 | 192.8 | 4507.3 | 191.9 | 0.8 | 4506.5 | 4.1% | 276.3 NE/1 | EN |
| *Phaner spp.* | 28696.1 | 23660.9 | 5720.2 | 22975.9 | 685.0 | 5035.2 | 82.5% | 1.4 E/2 | EN-VU |
| *Prolemur simus* | 10128.2 | 5048.4 | 7014.1 | 3114.1 | 1934.3 | 5079.8 | 49.8% | 72.7 NE/2 | CR |
| *Propithecus candidus* | 4916.0 | 1763.9 | 3330.8 | 1585.2 | 178.7 | 3152.1 | 35.9% | 220.0 NE/2 | CR |
| *Propithecus coquereli* | 5319.8 | 5101.3 | 941.5 | 4378.3 | 723.1 | 218.4 | 95.9% | 4.7 N/1 | EN |
| *Propithecus coronatus* | 6283.6 | 31560.3 | 724.7 | 5558.9 | 26001.5 | -25276.7 | 502.3% | 30.4 NE/1 | EN |
| *Propithecus deckenii* | 9356.3 | 12874.2 | 2440.6 | 6915.7 | 5958.5 | -3517.8 | 137.6% | 245.7 NE/2 | EN |
| *Propithecus diadema* | 14964.8 | 19077.5 | 2318.2 | 12646.6 | 6430.9 | -4112.7 | 127.5% | 87.4 NE/2 | CR |
| *Propithecus edwardsi* | 6334.9 | 4604.1 | 1800.3 | 4534.6 | 69.5 | 1730.8 | 72.7% | 9.6 N/2 | EN |
| *Propithecus perrieri* | 3780.9 | 4373.3 | 0.0 | 3780.9 | 592.4 | -592.4 | 115.7% | 4.0 N/2 | CR |
| *Propithecus tattersalli* | 848.8 | 800.0 | 91.8 | 757.0 | 43.0 | 48.8 | 94.2% | 3.6 NW/2 | CR |
| *Propithecus verreauxi* | 22951.1 | 26872.7 | 2753.4 | 20197.7 | 6674.9 | -3921.6 | 117.1% | 17.6 E/1 | EN |
| *Varecia rubra* | 3780.9 | 3904.2 | 2.5 | 3778.4 | 125.8 | -123.3 | 103.3% | 1.3 S/2 | CR |
| *Varecia variegata* | 29985.1 | 18785.5 | 11979.8 | 18005.3 | 780.2 | 11199.6 | 62.6% | 125.2 NE/2 | CR |
